# Rotavirus Outbreak Traced in Clinical, Environmental, and Food Matrices: Whole Genomic Characterization of an Equine-like G3P[8] Genotype

**DOI:** 10.1007/s12560-026-09678-2

**Published:** 2026-01-31

**Authors:** Fernanda Marcicano Burlandy, Bruna Campestrini Casarin, Meylin Bautista Gutierrez, Carina Pacheco Cantelli, Shênia Patricia Corrêa Novo, Rosane Maria Santos de Assis, Bruna Candia Piccoli, Amanda Haisi, Anelise da Costa Daneli, Roberta Danieli Marchesan, Julia Kuhns, João Pessoa Araújo Junior, Lílian Borges Teixeira, Alexandre Vargas Schwarzbold, Priscila de Arruda Trindade, Marize Pereira Miagostovich, Tulio Machado Fumian

**Affiliations:** 1 Laboratório de Virologia Comparada e Ambiental, Instituto Oswaldo Cruz, Fiocruz, Av. Brasil, 4365 – Pav. Hélio & Peggy Pereira, Rio de Janeiro, RJ 21040-900 Brazil; 2 Laboratório de Biologia Molecular e Bioinformática Aplicadas a Microbiologia Clínica, Departamento de Análises Clínicas e Toxicológicas, Universidade Federal de Santa Maria, Rio Grande do Sul, Brazil; 3 Instituto de Biotecnologia, Universidade Estadual Paulista - Campus de Botucatu, São Paulo, Brazil; 4 Departamento de Clínica Médica, Universidade Federal de Santa Maria, Rio Grande do Sul, Brazil; 5 Centro Estadual de Vigilância em Saúde, Rio Grande do Sul, Brazil

**Keywords:** Rotavirus A, Outbreak, Clinical samples, Water, Food, Genotyping, Acute gastroenteritis, Equine G3P[8] DS-1-like

## Abstract

The emergence of new viral strains has been frequently associated with the onset of outbreaks in different epidemiological settings. This study investigated an acute gastroenteritis (AGE) outbreak, which occurred at a university campus in southern Brazil, in 2024, affecting approximately 30–40% of undergraduate students, with over 400 reported cases. The objectives were to describe the epidemiological and molecular features of the outbreak, identify the probable transmission route, and assess the genetic relatedness of the detected strain to recently circulating rotavirus A (RVA) lineages. Clinical, environmental, and food samples were analysed for RVA and norovirus genogroup (G) I and GII. RT-qPCR detected RVA in the three specimen types with concentrations reaching 2.4 × 10^8^ genome copies (GC)/g in faeces, 3.5 × 10^2^ GC/g in food and 5.5 log₁₀ GC/l in water. Genotyping revealed an equine-like G3P[8] strain in clinical samples with whole genome sequencing establishing a DS-1-like backbone; G3-P[8]-I2-R2-C2-M2-A2-N2-T2-E2-H2. Sequencing data of the VP6 gene of RVA detected in one of the environmental samples confirmed the same genotype (I2), consistent with the genotype found in the clinical stool samples. The detection of RVA in multiple environmental and food specimens suggests that the possible source of contamination was associated with water supply systems. Phylogenetic analyses showed that the outbreak strain clustered with equine-like G3P[8] lineages. These findings highlight the importance of integrating molecular surveillance and environmental monitoring to trace viral transmission pathways and detect the emergence of RVA strains in community outbreaks.

## Introduction

*Rotavirus alphagastroenteritidis* (formerly Rotavirus A; RVA) is a member of the *Sedoreoviridae* family and belongs to the *Rotavirus* genus. Even after mass rotavirus vaccination programs worldwide, that significantly reduced disease burden in many countries (Burnett et al., 2020), RVA remains the most common and significant cause of severe acute gastroenteritis (AGE) among infants and young children (Cohen et al., 2022; Troeger et al., 2018).

The virion is a triple-layered, non-enveloped icosahedral particle comprising 11 segments of double-stranded RNA that encode six viral structural proteins (VP1, VP2, VP3, VP4, VP6, and VP7) and six non-structural proteins (NSP1, NSP2, NSP3, NSP4, NSP5/6). Traditionally, RVA were classified in a binary system into G (glycoprotein) and P (protease-sensitive) genotypes, based on the nucleotide sequence of the genomic segments that encoded the structural proteins VP7 and VP4, respectively. However, to improve understanding of rotavirus evolutionary dynamics - including reassortment events, point mutations, interspecies transmission, and genetic drift/shift within the segmented genome - a classification system based on the genetic constellation of all eleven gene segments was introduced (Matthijnssens et al., 2008). Identification is based on the nucleotide identity cutoff values of the genes that code for the proteins: VP7-VP4-VP6-VP1-VP2-VP3-NSP1-NSP2-NSP3-NSP4-NSP5/6, such as Gx-P[x]-Ix-Rx-Cx-Mx-Ax-Nx-Tx-Ex-Hx (Matthijnssens et al., 2008b, 2011). Human rotavirus genomes are further categorized into three primary genotypic constellation backbones: Wa-like (I1-R1-C1-M1-A1-N1-T1-E1-H1), DS-1-like (I2-R2-C2-M2-A2-N2-T2-E2-H2), and AU-1-like (I3-R3-C3-M3-A3-N3-T3-E3) (Cao et al., 2023).

To date, 42 G- and 58 P- genotypes capable of infecting humans and animals have been described (https://rega.kuleuven.be/cev/viralmetagenomics/virus-classification/rcwg). However, most global cases of human disease are caused by a limited set of combinations (including G1P[8], G2P[4], G3P[8], G4P[8], G9P[8], and G12P[8]). Although binary genotyping allows numerous G[P] combinations, the six predominant circulating genotypes associated with human infection are G1, G3, G4, G9, and G12 in combination with P[8], and G2P[4], accounting for over 90% of infections globally (Crawford et al., 2017 Dóró et al., 2014).

Approved rotavirus vaccines (such as Rotarix^®^, RotaTeq^®^, Rotavac^®^ and Rotasiil^®^) have been formulated to protect against the predominant genotypes but also offer cross-protection against genotypes not present in the vaccine formulations. (Wang et al., 2021; World Health Organization. (‎^2009^)‎. Rotavirus vaccines : an update. Weekly Epidemiological Record = Relevé épidémiologique hebdomadaire,* 84 (‎51–52)‎*,* 533–540.* https://iris.who.int/handle/10665/241492, s. d.).

Since immunity to severe rotavirus gastroenteritis is acquired early in life, vaccination is administered within the first year; older children and adults often experience mild or asymptomatic infections(Cardemil et al., 2012; Iijima et al., 2006). The occurrence of severe AGE in adults, as observed in this and other outbreaks, may be attributed to the emergence of novel viral strains or variants resulting from genetic mutation, reassortment, or recombination and may result in rapid spread among susceptible populations and sometimes caused symptomatic outbreaks, such as the spread of equine-like G3P[8] rotavirus strains (Karataş et al., 2025; Luchs et al., 2019; Ma et al., 2025; Niendorf et al., 2020).

This study aimed to investigate an RVA-associated AGE outbreak that occurred in a university community, in southern Brazil. The investigation sought to elucidate the epidemiological and molecular characteristics of the outbreak and to establish potential links between human cases and environmental sources. For this purpose, clinical, environmental, and food samples collected during the epidemiological investigation were analyzed to identify the source and transmission route of infection. Furthermore, whole-genome and phylogenetic analyses were conducted on a subset of clinical samples to characterize the equine DS-1-like G3P[8] strain.

## Materials and Methods

### Outbreak Description

In late September 2024, an AGE outbreak occurred at a federal university campus in Santa Maria city, Southern Brazil, affecting primarily students living in the university’s dormitories. Preliminary reports from the Epidemiological Surveillance Agency documented more than 200 symptomatic cases, including 30 laboratory-confirmed rotavirus A (RVA) infections, identified through rapid antigen detection by enzyme-linked immunosorbent assay (ELISA; Ridascreen Rotavirus, R-Biopharm, Germany). All preliminary assays were conducted at the Central Public Health Laboratory of the State of Rio Grande do Sul, following the diagnostic workflow established by the General Coordination of Public Health Laboratories as part of the rotavirus surveillance network.

Ten students required hospitalization due to symptom severity. On September 30th, the university implemented immediate containment measures, including a 48-hour suspension of in-person classes and closing of restaurants for deep cleaning to reduce transmission risk. Symptomatic students were instructed to avoid campus and seek medical evaluation.

At the same time, public health authorities began an investigation of the outbreak, collecting food samples from the cafeteria and water samples from different areas of the campus as the epidemiological links suggested potential transmission sites. They also conducted interviews, inter-institutional meetings and developed educational materials to contain the outbreak spread. Enhanced disinfection protocols were applied to high-contact surfaces in restaurants and dormitories, with particular focus on shared bathrooms and common areas where transmission appeared most active. On October 1st, the institution established an online syndromic surveillance system that recorded 150 symptomatic reports within 48 h. By October 3rd, Municipal Health authorities observed a significant decline in new cases permitting resumption of academic activities, with reinforced preventive measures including mandatory hand sanitizer use, prohibition of item sharing, and isolation protocols for symptomatic individuals. On October 4th, no new severe cases were reported, indicating successful outbreak containment, and health authorities declared the event under control.

### Laboratory Investigation

#### Ethical Statement

This study is currently approved by the Ethics Committees of the Oswaldo Cruz Foundation (FIOCRUZ) and Federal University of Santa Maria, Brazil (Approval number: CAAE 94144918.3.0000.5248 and CAAE 76166623.0.0000.5346, respectively) and was conducted according to the guidelines of the Declaration of Helsinki.

Eleven samples of the outbreak were sent to the Regional Rotavirus Reference Laboratory (RRRL) for virus quantification and molecular characterization. The RRRL is part of the national network for rotavirus surveillance, which is coordinated by the General Coordination of Public Health Laboratories within the Brazilian Ministry of Health (MoH). The AGE aetiology surveillance is performed through a hierarchical network in which samples are provided by medical request in hospitals and health centers, monitored by the Brazilian Unified Health System (SUS).

#### Laboratorial Methodologies

Clinical stool samples were obtained from symptomatic patients who required medical attention at the university hospital. Samples were analysed and screened anonymously, and patients’ data were maintained securely.

**Fig. 1:**
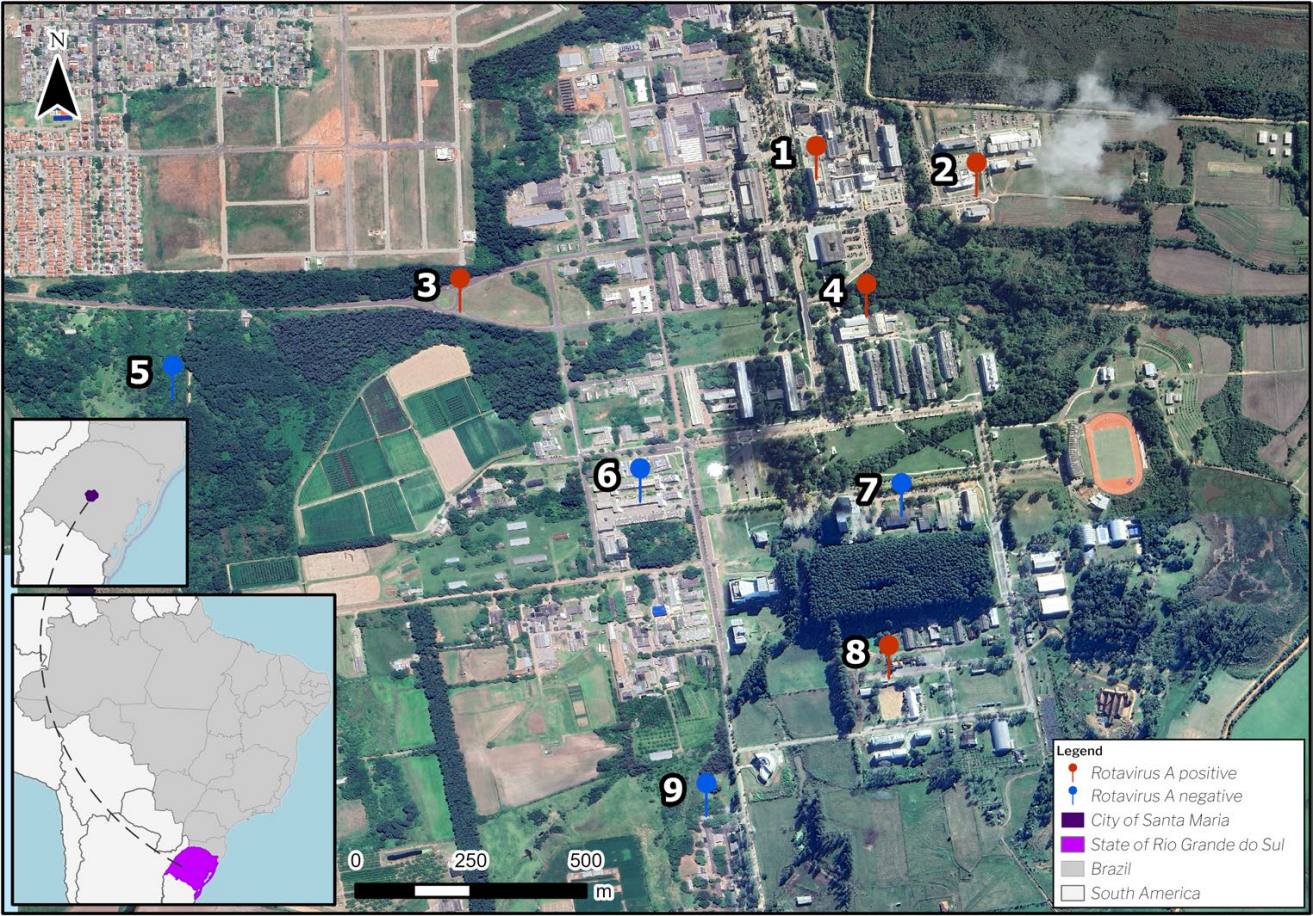
Geographic map of Brazil highlighting the state of Rio Grande do Sul (Southern region) and Santa Maria city, showing sampling points (1–9). Red pins indicate RVA-positive samples and blue pins indicate RVA-negative samples. Sampling sites: (1) University Hospital, (2) Building 28, (3) Botanical Garden Road, (4) University Refectory, (5) Botanical Garden, (6) Center for Social and Human Sciences, (7) University Rectory, (8) Horse-Riding Facility, (9) Veterinary University Hospital

Food and water samples potentially linked to cases of AGE reported by the students were collected by the Environmental Surveillance Service of the University from different settings (Fig. 1; Table 1). Five suspected food items were collected, including: grated zucchini (*n* = 2), raw tomato cut into slices (*n* = 2), cooked vegetable salad with or without added fruit pieces (*n* = 2), potato-mayonnaise salad (*n* = 2), and sliced red cabbage (*n* = 1). Samples were aliquoted (five aliquots of approximately 125 g of each), stored in original plastic packaging, and shipped under refrigeration to the laboratory.

**Table 1:** Description and results of rotavirus detection in food and water samples associated with outbreak

Sample type	Collection date	Food and water sample description	Additional information about the sampling	RVA Detection; Viral load GC/g
		Grated zucchini		negative
		Raw tomato (cut into slices)	served at lunch on september 28th on university refectory	negative
		Cooked vegetable salad		negative
		Potato mayonnaise (ready-to-eat)		negative
		Raw tomato (cut into slices)		negative
		Potato mayonnaise (ready-to-eat)	served at dinner on september 28th on university refectory	negative
		Grated zucchini		negative
Food		Sliced red cabbage		negative
	Sep 30th, 2024	Cooked vegetable salad		3.52 × 10^2^ GC/g
		Cooked vegetable salad - aliquot 1		negative
		Cooked vegetable salad - aliquot 2	served at lunch on september 30th on university refectory	2.40 × 10^2^ GC/g
		Cooked vegetable salad - aliquot 3		negative
		Cooked vegetable salad - aliquot 4		negative
		Cooked vegetable salad - aliquot 5		9.41 × 10^1^ GC/g
	Oct 02nd, 2024	Faucet after the reservoir	Water reservoir of the central campus	5.23 Log _10_ GC L^− 1^
		Water intake point	Post-desinfection tap	5.09 Log _10_ GC L^− 1^
		Faucet after the reservoir	Post-filter taps before drinking fountains at the university refectory	5.50 Log _10_ GC L^− 1^
			Water well at the university rector’s building	negative
Water			Artesian well at the veterinary university hospital	negative
			Water well at the university’s horse-riding facility	4.99 Log _10_ GC L^− 1^
			Artesian well of one of the university building 28	4.82 Log _10_ GC L^− 1^
			Artesian well at the cloverleaf road botanical garden	5.00 Log _10_ GC L^− 1^
	Nov 04th ,2024	Water intake point	Artesian well at the botanical garden	negative
			Artesian well at the center for social and human sciences	negative
			Artesian well at the transport sector	negative
			Water reservoir outlet of the university rector’s building	negative
	Nov 11th, 2024		Drinking fountain at the rector’s building	negative
	Nov 18th, 2024		Water well of the university’s waste treatment plant	negative

* Disinfection was performed by chlorination, as required by local drinking water regulations

Water samples (2 L each) were collected from university facilities, totalling 14 samples, comprising five treated and nine untreated water sources. Treated samples were obtained from reservoir (*n* = 2), post-reservoir faucet (*n* = 2), and intake point (*n* = 1) sources. Untreated samples were obtained from artesian wells which served as additional intake points. All samples were transported under refrigeration in sterile bottles for viral analysis.

#### Food Viral Concentration Method

Viral concentration from food matrices was conducted in accordance with the ISO 15216-1:2017 (ISO 15216-1:^2017^. Microbiology of Food a Chain—Horizontal Method for Determination of Hepatitis A Virus and Norovirus in Food Using Real-Time RT-PCR—Part 1: Method for Quantification. International Organization for Standardization: Geneva, Switzerland, 2017; *Volume 2017*,* pp. 1–48.*, s. d.) standard protocol for soft fruits and leafy, stem, and bulb vegetables, with minor modifications. Composite samples (25 g) were prepared by pooling five 5 g aliquots. Each pooled sample (25 g) was transferred to a sterile 400 mL polypropylene stomacher bag and inoculated with 10⁷ genome copies (20 µL) of the mengovirus strain (MgV vMC0) as an internal process control, following the approach described by Cantelli et al. (2024). Samples were eluted with 33 mL of an alkaline buffer (pH 9.5; 100 mM Tris-HCl, 50 mM glycine, 1% beef extract) and agitated for 20 min at ambient temperature. The eluates were recovered and subjected to ultracentrifugation at 10,000 × g for 30 min at 4 °C. The resulting supernatants were transferred to clean tubes, adjusted to pH 7.2, and supplemented with approximately 0.5 volumes of 33% (w/v) polyethylene glycol (PEG) and 1.5 N NaCl. The mixtures were gently agitated on ice for 1 h and subsequently ultracentrifuged at 10,000 × g for 30 min at 4 °C. The final viral pellet was resuspended in 700 µL of phosphate-buffered saline (PBS, pH 7.4) and stored for subsequent RNA extraction.

#### Water Viral Concentration Method

Viral particles from water samples were concentrated using the adsorption-elution method with positively charged membranes, followed by ultrafiltration through Amicon Ultra 50 kDa^®^ filters (Millipore, Tokyo, Japan), in accordance with ISO 15216-1:2017 standards for water samples. The bacteriophage PP7, kindly provided by Dr. Verônica Rajal, was used as internal control process and produced as following: *Pseudomonas aeruginosa* (ATCC 15692-B2) was grown on nutrient agar at 37 °C for 18–24 h. A loopful of colonies was inoculated into 10 mL nutrient broth (8 g/L) and incubated overnight at 37 °C. Subsequently, 1 mL of PP7 stock (≈ 10⁹ PFU/mL) was added to each culture and incubated for 18 h for viral replication. The lysates were filtered through 0.22 μm filters, and the resulting filtrate (10⁷–10⁹ PFU/mL) was aliquoted and stored at 4 °C (Rajal et al., 2007). Bacteriophage PP7 suspensions (10^7^GC/µL) were inoculated in all water samples before virus concentration.

#### RNA Extraction

RNA was extracted from 140 µL of clarified stool suspension (10% *w*/*v*) using the QIAcube automated system and the QIAamp Viral RNA Mini kit (both from QIAGEN, Valencia, CA, USA). Viral RNA extracted was eluted in 60 µL of the elution buffer (AVE) and immediately stored at -80 °C until the molecular analysis. RNAse/DNAse-free water was used as a negative control in each extraction procedure.

For food and water samples, RNA was extracted using the silica-magnetic bead extraction method MDX^®^ DNA and RNA Pathogens kit using the Extracta Loccus^®^ equipment (Biotech Research Supplies, São Paulo, Brazil), according to the manufacturer’s instructions. For environmental samples, 300 µL of viral concentrate was processed, and RNA was eluted in 80 µL of elution buffer.

#### Viral Analysis

All samples were investigated for the presence of RVA and norovirus GI and GII. For RVA, primers (NSP3F/R) and probe (NSP3p) were used to amplify the conserved NSP3 gene (Zeng et al., 2008). For the detection of noroviruses GI and GII, primer pair (COG1F/R; COG2F/R) and probes (RING1c and RING2) targeting the ORF1/2 overlapping region were used (Hill et al., 2010; Kageyama et al., 2003). All runs included also a standard curve with serial dilutions (10^1^–10^7^) of double-stranded DNA fragments (gBlock^®^ Gene Fragment, Integrated DNA Technologies, Iowa, USA) containing the target region to ensure the correct interpretation of the results throughout the study. Viral loads were expressed as genome copies per gram (GC/g) of stool.

For clinical stool samples, the One-step RT-qPCR was used with SuperScript III Platinum One-Step Quantitative RT-PCR kit (Invitrogen, Carlsbad, CA, USA) and 5 µL of extracted nucleic acid for a final volume of 25 µL. Samples that showed a Ct value ≤ 33 with a characteristic sigmoid curve were considered positive.

For food and water samples, viruses’ detection included an initial step of cDNA synthesis using either the Superscript III reverse transcription kit (Invitrogen, Carlsbad, CA) with random primers, or the High-Capacity cDNA Reverse Transcription Kit (Applied Biosystems Massachusetts, USA). The cDNA generated (5 µL) was used for RVA and norovirus detection with primers and probes as described above, using the TaqMan Universal Master Mix PCR Kit (ThermoFisher Scientific, Invitrogen Division, Carlsbad, CA, USA) in a 25 µL final volume. Additionally, MgV vMC_0_ and PP7 were detected from those environmental samples using primers and probes as described previously (Pintó et al., 2009; Rajal et al., 2007).

Positive controls (containing RNA extracted from stool suspensions) and negative controls (DNAse and RNAse-free water) were included in all qPCR assays. The viral nucleic acid obtained from each food and water sample was tested (pure and 10-fold diluted, in duplicate each), totaling four qPCR reactions per sample. MgV vMC_0_ and PP7 recovery rate percentages were calculated for each mean replicate sample (pure or 10-fold diluted) as described in ISO15216-1:2017. The food and water samples were considered positive when at least the duplicate analyzed was detected at the cycle threshold (Ct) < 45.

#### RVA Genotyping and Genomic Characterization

RVA-positive clinical stool samples were initially G and P genotyped using a one-step multiplex RT-PCR. The reactions were performed using the Qiagen One-Step RT-PCR kit (QIAGEN, Valencia, CA, USA) and forward conserved primers VP7uF or VP4uF, as well as specific reverse primers for G types G1, G2, G4, G6, G3, G9, and G12 or P types P[4], P[6], P[8], P[9], and P[10] as recommended by the Centers for Disease Control and Prevention, USA. The G and P genotypes were assigned based on different amplicon sizes and base pairs (bp) using agarose gel analysis (Esona & Gautam, 2015; Gentsch et al., 1992; Iturriza-Gómara et al., 2001).

RVA sequencing from food and water samples was performed using a semi-nested PCR with primers targeting the partial VP6 gene according to published methods (Iturriza Gómara et al., 2002; Matthijnssens et al., 2008a). The first round (50 µL total volume) contained 10 µL cDNA, while the second round was performed using 5 µL of the primary amplicon. The Platinum™ Taq DNA Polymerase kit (Invitrogen, Carlsbad, CA) was used for both amplification reactions.

The generated amplicons were purified using the QIAquick Gel Extraction Kit (QIAGEN, Valencia, CA, USA) and sent to the FIOCRUZ Institutional Platform for DNA sequencing (PDTIS). Sequencing reactions were run using both forward and reverse primers on an ABI Prism 3730xl genetic analyzer (Applied Biosystems, Foster City, CA, USA). Chromatogram analysis and RVA consensus sequences were obtained using BioEdit 7.7.1 Sequence Alignment Editor and Geneious Prime 2021.1.1 (Biomatters Ltd, Auckland, New Zealand). The assembled sequences were compared with sequences of known RVA genotypes using the NCBI BLASTN tool or were submitted to the ViPR rotavirus A genotype determination tool.

#### Whole-Genome Sequencing

Four RVA-positive clinical samples with low Ct values (< 25) were selected to characterize the 11 genomic segments by metagenome sequencing. The extracted RNA was treated with TURBO DNase Kit (Invitrogen, Carlsbad, CA) performing the DNase inactivation step according to manufacturer’s instructions. To reduce host and microbiome interference, a ribosomal RNA (rRNA) depletion step was performed with QIAseq *FastSelect Epidemiology Kit* (QIAGEN, Valencia, CA, USA). Amplification was performed using the Sequence-Independent, Single-Primer Amplification (SISPA) method (Lewandowski et al., 2019), with the following modification: Q5^®^ High-Fidelity 2X Master Mix (New England Biolabs, Ipswich, MA, USA) was used for both second-strand cDNA synthesis and subsequent PCR amplification. PCR products were quantified in a Qubit 4 fluorometer (Qiagen). Sequencing reactions were run using nanopore sequencing with the Ligation Sequencing Kit (SQK -LSK109) and Native Barcoding Expansion Kit (EXP-NBD104, EXP-NBD114), using the R9.4.1 FLO-MIN106D Flow Cell on a MinION MK1c device for 48 h (ONT, Oxford, UK). To obtain a complete genome and to validate the data generated by nanopore sequencing, the samples were also sequenced using an Illumina iSeq 100 device (Illumina, Inc., San Diego, CA, USA). This process utilized the Illumina DNA Prep kit along with a V2 cartridge.

#### Genome Assembly, Taxonomic Classification, and Genotyping

Raw data from the MinION sequencer was collected using MinKNOW 5.5.3 High-accuracy base-calling. Demultiplexing, including barcode and adaptor trimming, were performed using Dorado v0.8.3. Quality assessment of the raw data and trimming was carried out with Fastp v0.23.4 (Chen et al., 2018). The trimmed FASTQ files were mapped to the human reference genome (GenBank accession number: GCF_000001405.40) to remove host-derived reads. The unmapped reads were then subjected to taxonomic classification using Kraken2 v2.1.3 (Wood et al., 2019) with the PlusPF database. Reads classified as viral were extracted using the extract_kraken_reads.py function from KrakenTools and subsequently used for RV genome assembly (Lu et al., 2022).

For the Illumina sequencing data, base-calling and demultiplexing were performed using Illumina’s BaseSpace platform. The quality of the raw data was assessed using FastQC v.0.12.1 (Babraham Bioinformatics). Adapters and low-quality reads were removed using Trimmomatic v0.39 (Bolger et al., 2014). Short reads were then assembled de novo using SPAdes v3.13.1 (Bankevich et al., 2012).

For both Illumina and MinION sequencing datasets, SISPA primers were removed using Cutadapt (Martin, 2011). The 11 genomic segments of RV were assembled using Geneious Prime version 2023 by mapping the reads to the reference genome assembly ASM265967v1 (GenBank accession numbers: JN129122, JN129094, JN129108, JN129052, JN129066, JN129080, JN128982, JN128996, JN129010, JN129024, JN129038). A consensus sequence was generated from both datasets, with the Illumina-derived consensus serving as a scaffold to map the long-read data using the Geneious mapper, enabling accurate reconstruction of the 11 RVA segments. Open reading frames (ORFs) were subsequently identified using the ORF Finder tool (https://www.ncbi.nlm.nih.gov/orffinder/, accessed on 18 March 2025). The assembled sequences were submitted to the ViPR Viral Subspecies Classification tool on BV-BRC for genotyping.

#### Phylogenetic Analysis

Chromatogram analysis and consensus sequences were obtained using the Geneious Prime software (Biomatters Ltd., Auckland, New Zealand). The genotypes of VP7 and VP4 were identified using the Rotavirus A Genotyping Tool Version 0.1 (https://mpf.rivm.nl/mpf/typingtool/rotavirusa/, accessed on 25 June 2025). The Basic Local Alignment Search Tool (BLAST) server was also used as a complementary tool for genotype identification. Multiple alignments of the sequences were performed using the CLUSTAL W program in MEGA 11 v. 11.08.

Phylogenetic analysis was performed using the Maximum likelihood method (MLM) in MEGA 11 software v. 11.08. The selected best-fit evolutionary model for the data set via Tamura 3-parameter (T92 + G + I; VP7 gene), (T92 + G; VP4, NSP2 and NSP5 genes), (T92 + I; VP1, VP6 and NSP4 genes) and (TN93 + G + I; VP2 and NSP3 genes). The trees were measured using the bootstrap tests with 1000 replicates. Bootstrap values above 85% are given at branch nodes. Nucleotide sequences obtained in this study were deposited in the GenBank database under the accession numbers (PV753008-PV753051).

## Results

### Epidemiological and Clinical Findings

**Fig. 2:**
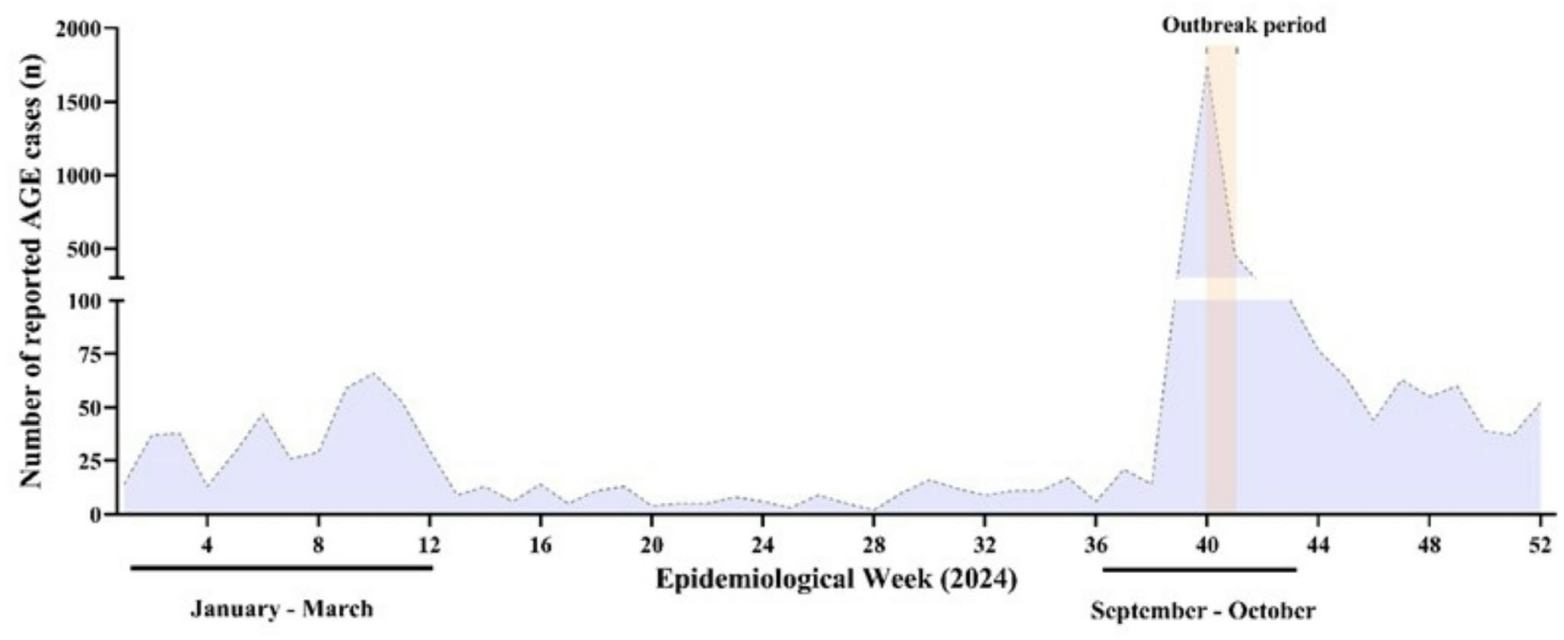
Distribution of acute gastroenteritis (AGE) cases along 2024 in Santa Maria city, Southern Brazil. The outbreak is indicated by the shaded area

Overall, 1,806 notifications were received: 467 confirmed cases (student or university staff presenting diarrhea (≥ 3 episodes in 24 h) and at least one additional symptoms - nausea, vomiting, abdominal pain, or fever, or individuals epidemiologically linked to a laboratory-confirmed RVA case), 227 discarded cases (individuals who not match the suspected case definition), and 1,112 probable cases (reports received during the outbreak period that matched the suspected case definition but could not be classified as confirmed or discarded due to incomplete information in the notification forms or medical records, and unsuccessful telephone contact attempts). In total, the outbreak affected approximately 30–40% of undergraduate students, with more than 400 AGE reported cases. Clinical manifestations included diarrhea (93.7% of cases), vomiting (81.2%), fever ≥ 38 °C (64.3%), and abdominal pain (76.5%) (Fig. 2).

### RVA Detection

During the outbreak, a total of 34 samples, including stool (*n* = 11), food (*n* = 9) and water (*n* = 14) samples, were analysed and 41.2% (14/34) of the specimens tested positive for RVA (Table 2). All samples were screened for norovirus GI and GII with negative results across all tested specimens. We detected RVA in 63.6% (7/11) of faecal samples with viral loads ranging from 4.4 × 10^6^ to 2.4 × 10^8^ GC/g of stool samples, with a median of 1.9 × 10^7^ GC/g. Additionally, RVA was detected in 11.1% (1/9) of food samples and in 42.9% (6/14) of water samples (Table 2).

**Table 2:** Type of tested and virus-positive samples through laboratory outbreak investigation

Samples type	RVA detection Pos/tested (%)	Viral load range	Genotyping RT-PCR	Whole genome constellation
Clinical	7/11 (63.6)	4.4 × 10^6^ – 2.4 × 10^8^ GC/g	G3[P8]	G3-P[8]-I2-R2-C2-M2-A2-N2-T2-E2-H2
Food	1/9 (11.1)	3.5 × 10^2^ GC/g	-	-
Treated water	3/6 (50)	5.1–5.5 log₁₀ GC/l	-	-
Untreated water	3/8 (37.5)	4.8–5 log₁₀ GC/l	I2	-

The extraction efficiency (mean ± standard deviation) of all processed water and food samples was calculated based on the recovery of the internal control PP7 (13.32 ± 14.18%) and MgV vMC_0_ (82.2 ± 26.7%). RVA was detected in three treated water samples, with viral loads ranging from 5.09 to 5.50 log₁₀ GC l⁻¹, and a mean of 5.27 log₁₀ GC l⁻¹ ± 0.21 (mean ± standard deviation). The highest detection rate (37.3%) and viral concentration were found in a water faucet located in the university dining hall (Table 1). Of the three untreated water samples, the viral load ranged from 4.8 to 5.0 log₁₀ GC l⁻¹, with a mean of 4.94 log₁₀ GC l⁻¹ ± 0.10 (mean ± standard deviation). The viral detection rates in these samples were similar, with the highest being 37.8%.

With regards to food samples, one cooked vegetable salad of the nine samples investigated tested positive for RVA (3.5 × 10^2^ CG/g, pool sample number id. 8276). Five aliquots from this pool were resampled (25 g of each), processed, and analyzed individually. Of the new analyses, two aliquots demonstrated positive results (2.4 × 10^2^ CG/g and 9.4 × 10^1^ CG/g), confirming the initial result.

### RVA G and P Genotyping and Genome Constellation

**Fig. 3:**
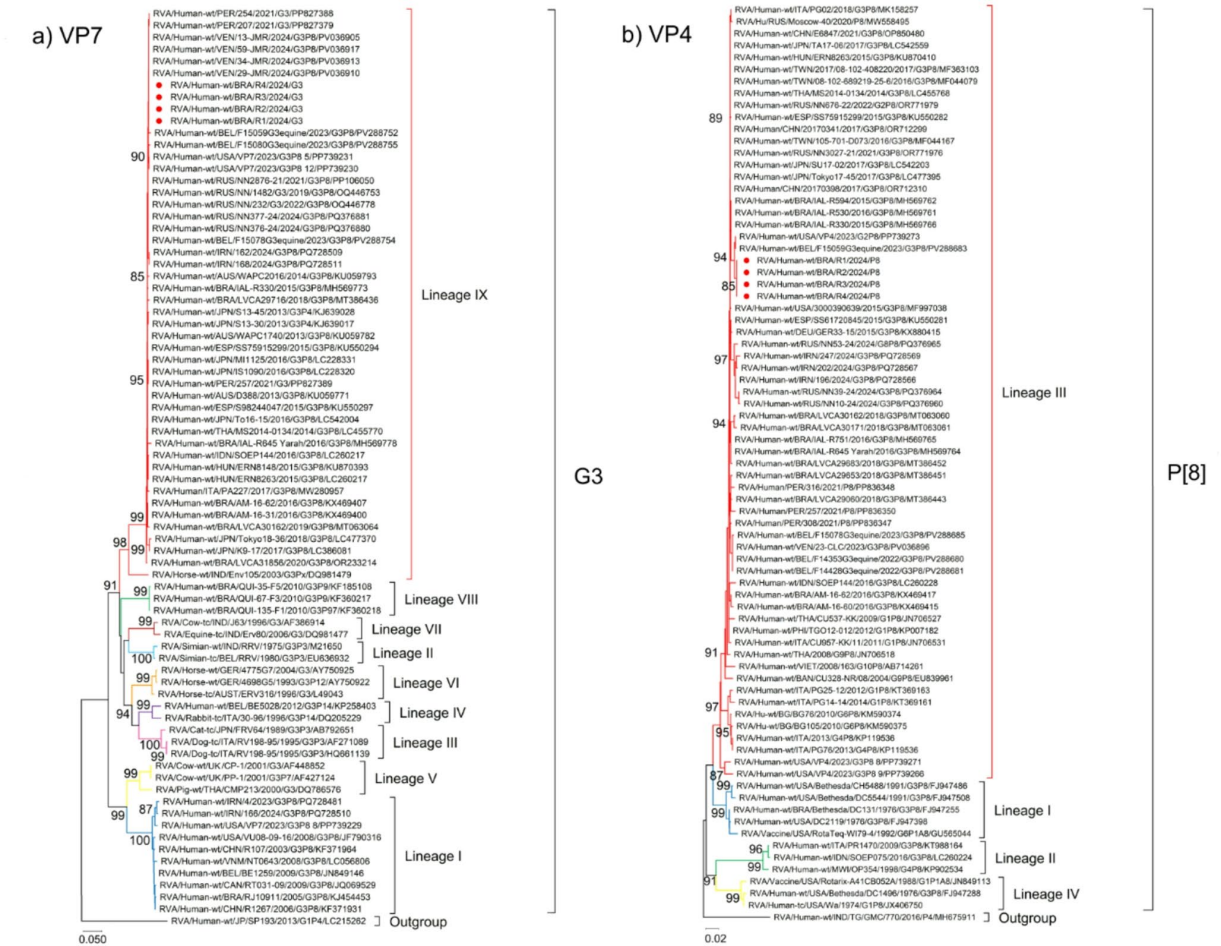
Phylogenetic analysis of G3P[8] rotavirus strains isolated from Brazilian patients during the outbreak. Reference strains were downloaded from GenBank and labeled with their respective accession numbers. Sequences obtained from our study (marked with a red circle) were labeled with viral species, origin, country, internal register number, year of collection, genotype and accession number (i.e. RVA/Human wt/BRA/LVCA29653/2018/G3P8/MT386451). Maximum-likelihood (ML) phylogenetic tree of G3 and P[8] were constructed based on the partial coding sequence of the VP7 (**A**) and VP4 (**B**) genes, respectively, with MEGA 11 software and bootstrap tests (1000 replicates), based on the Tamura 3-parameter (T92 + G + I; VP7 gene) and Tamura 3-parameter (T92 + G + I; VP4 gene). Bootstrap values above 85%, are indicated at each node

Concerning RVA molecular characterization, 100% (7/7) of positive fecal samples (Ct < 30) were amplified and successfully characterized for G and P as an equine G3P[8] genotype (Fig. 3).

**Fig. 4:**
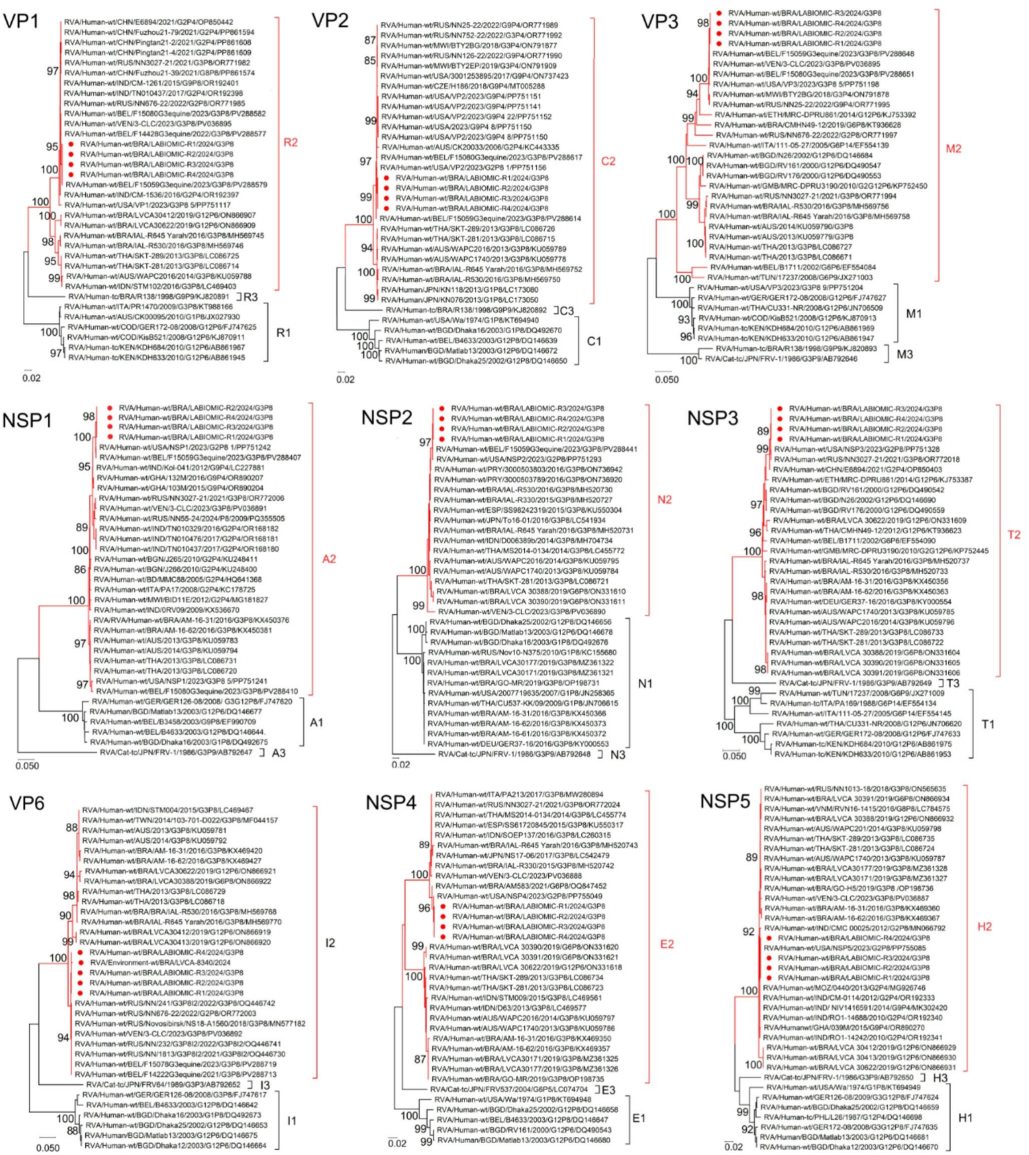
Phylogenetic analyses based on VP1-VP3, VP6, NSP1-NSP5 nucleotide (nt) sequences of circulating Brazilian G3P[8] strains in 2024. The strains obtained in this study (marked with a filled red circle) and reference strains that were downloaded from GenBank were both labeled as follows: RV group, species of origin, country, common name, year, G and P genotype and access number. Maximum-likelihood (ML) phylogenetic trees were constructed with MEGA 11 software and bootstrap tests (1000 replicates) based on the Tamura 3-parameter models T92 + G (NSP2, NSP5), T92 + I (VP1, VP6, NSP1, NSP3, NSP4) and TN93 + G + I (VP2, VP3). Bootstrap values above 85% are given at branch nodes

To investigate the gene constellation of the Brazilian strain circulating in the outbreak, we selected four samples based on viral load, and a whole-genome analysis was performed. The nucleotide sequence of the 11 segments revealed a DS-1-like genotype constellation of G3-P[8]-I2-R2-C2-M2-A2-N2-T2-E2-H2. For environmental samples, it was possible to characterize only one partial gene (VP6) segment due to the low viral load. The genotype detected from the one water sample (sample number id. 8340) was I2 and nucleotide and amino acid sequence matched 99.7% and 100%, respectively, compared to the VP6 sequences obtained from clinical samples (Fig. 4).

#### Genetic Analysis of Structural Genes - VP7 and VP4

Phylogenetic analysis of the VP7 gene demonstrated that sequences from the outbreak grouped into lineage IX, corresponding equine-like G3P[8] genotype, with > 99% nucleotide identity to sequences from Belgium, Venezuela, and Peru (2021–2024). Sequences from the USA, Russia, and Ireland grouped in different clusters with 89.9–92% nucleotide identity (Fig. 3A).

Regarding the VP4 gene, the study sequences clustered into lineage III, showing 99.7–99.8% identity with P[8] sequences from Belgium and the USA (2023). Other P[8] sequences in the same lineage segregated in different branches with 98.9–99.0% identity to sequences from Brazil, USA, Spain, and Germany (Fig. 3B).

#### Genetic Analysis of Structural Genes - VP1, VP2, VP3 and VP6

Phylogenetic analysis of structural genes (VP1–VP3; VP6) showed high nucleotide identity with recent international strains: VP1 and VP3 (> 99%) with Belgium (2023), VP2 (> 97.6%) with USA (2023), and VP6 (99.4–99.6%) with Russian sequences (Fig. 4).

#### Genetic Analysis of Non-Structural Genes - NSP1 To NSP5

Non-structural genes (NSP1–NSP5) also exhibited high similarity with global strains: NSP1 and NSP2 (> 99.6%) with USA and Belgium sequences (2023), NSP3 (99.7–99.8%) with Russia and USA, NSP4 (99.7–99.8%) with USA (2023) and Brazil (2021), and NSP5 (> 99%) with USA (2023) and Indonesia (2012), respectively (Fig. 4).

## Discussion

This study investigated an outbreak associated with detection of an equine-like G3P[8] RVA among students at a university campus in Southern Brazil, 2024. The integrated investigation of multiple clinical and environmental samples suggested a probable waterborne transmission route, as RVA was detected in six water samples from the university’s water wells.

This outbreak was initially confirmed based on clinical and epidemiological criteria when more than 200 symptomatic students were identified, with 30 laboratory-confirmed RVA infections, corresponding to 15% of the total reported cases. The high attack rate (30–40% of the undergraduate population) and the rapid onset of symptoms including diarrhoea, vomiting, fever, and abdominal pain strongly support the occurrence of a well-defined outbreak. Epidemiological links between cases, combined with environmental and food investigations, suggest a common source of infection, probable related to the water supply systems in university facilities. Despite the large number of people affected, the institution’s rapid response, including timely class suspension and environmental interventions, proved critical in limiting outbreak duration to under one week.

Based on the complete genome constellation analysis, the detected equine-like G3P[8] strains were identified as DS-1-like backbone (genogroup 2). In addition, RVA was detected and sequenced from one water sample, with the VP6 gene confirming the same genotype I2, consistent with the genotype found in the clinical stool samples supporting the link between clinical and environmental circulation of RVA during the studied period. Amplification and sequencing of the VP6 gene, a conserved region of the RVA genome, is usually employed to confirm the detection of RVA in environmental samples (Lanzarini et al., 2023).

The detection of RVA in both clinical and water samples, along with comparable viral loads in treated and untreated water samples, suggests contamination within the water distribution system.

The quantification of RVA in one food sample reinforces the hypothesis of environmental transmission indicating that food was unlikely the primary vehicle of transmission. Although it was not possible to determine whether any of the positive water samples supplied the restaurant facility, contamination may have resulted from multiple factors. The viral load detected in the cooked salad suggests possible contamination during food handling or preparation. It is well known that inadequate hand hygiene, improper cleaning of food-contact surfaces, and insufficient temperature control are key factors facilitating viral spread in food service settings (Alidjinou et al., 2019; Omatola & Olaniran, 2022). However, the potential contamination of the university’s main water reservoir should also be considered. The rapid decline in case numbers following sanitary measures implementation further supports a point-source, waterborne outbreak.

The absence of norovirus GI and GII, in the tested samples supports RVA as the primary causative agent, since norovirus is the most common etiological agent of AGE in adults being responsible for a significant proportion of reported AGE outbreaks globally (Barclay et al., 2021; Raymond et al., 2022; Rumble et al., 2017; Saupe et al., 2021; Wikswo et al., 2022). In Brazil, we recently described two norovirus foodborne outbreaks related to uncommon genotypes (Burlandy et al., 2025); (Fumian et al., 2021). However, the fact that other viral or bacterial pathogens were not tested means that co-infections or additional contributing agents cannot be entirely ruled out, representing a potential limitation of this study. While AGE outbreaks are a common public health concern, those caused by RVA are relatively uncommon, particularly in adult populations. However, RVA outbreaks have been reported including a university hospital in Germany (2016) where rotavirus G2P[4] was detected in 32 adult cases across nine different wards (Niendorf et al., 2020). In King County, Washington, a G9P[4] was the predominant genotype among adults between January and June 2023 (Ma et al., 2025). In Brazil, following the RVA vaccine implemented into the National Immunization Program in 2006, G2P[4] emerged as the dominant strain, alongside increased detection of G3P[8] and G12P[8] genotypes (Carvalho-Costa et al., 2019). However, since 2016, the equine-like G3P[8] strain has been detected in cases of diarrhoea in several regions of the country (Luchs et al., 2019).

In this study, nucleotide sequencing of the VP7 and VP4 genes demonstrated that the isolates clustered into the G3 lineage IX and the P[8] lineage III, with a DS-1-like genomic backbone, that showed a close genetic relationship with equine-like G3 human strains (Guerra et al., 2016; Gutierrez et al., 2024; Luchs et al., 2019). The whole-genome analysis revealed that this equine-like G3P[8] strain exhibited genetic differences relative to previously Brazilian G3 strains identified by our group from diarrheal cases surveillance (Gutierrez et al., 2020, 2024). Phylogenetic comparison also demonstrated that these Brazilian G3P[8] DS-1-like strains are closely related to recent equine-like G3 lineages circulating in Belgium, Venezuela, Peru, Russia, and the USA (Karataş et al., 2025; Ma et al., 2025; Morozova et al., 2023; Vizzi et al., 2025).

The first reports of sporadic cases of the equine-like G3P[8] DS-1 strain were from Asia (Komoto et al., 2016; Malasao et al., 2015) and Australia (Cowley et al., 2016) during 2013 to 2015 and then emerged as a predominant genotype in several countries worldwide, including Asia (Pakistan, Indonesia, Japan), South America (Brazil) and Europe (Germany, Spain) (Guerra et al., 2016; Komoto et al., 2016; Luchs et al., 2019; Medici et al., 2016; Pietsch & Liebert, 2018; Umair et al., 2018).

In conclusion, laboratory findings provide strong evidence that the acute gastroenteritis outbreak among university students was caused by waterborne transmission of RVA equine-like G3P[8] genotype. This underscores the public health importance of continuous water quality surveillance, the integration of molecular diagnostic tools in outbreak investigations, and the prompt implementation of sanitary interventions to prevent similar events in institutional settings. Ongoing monitoring and genomic characterization of circulating RVA strains remain essential for the early detection of emerging variants and the mitigation of future outbreaks.

## Data Availability

Data will be made available on request.

## References

[CR1] Alidjinou, E. K., Sane, F., Firquet, S., Lobert, P. E., & Hober, D. (2019). Resistance of enteric viruses on fomites. *Intervirology*, *61*(5), 205–213. 10.1159/000448807 10.1159/000448807 PMC7179519 28614823

[CR2] Bankevich, A., Nurk, S., Antipov, D., Gurevich, A. A., Dvorkin, M., Kulikov, A. S., Lesin, V. M., Nikolenko, S. I., Pham, S., Prjibelski, A. D., Pyshkin, A. V., Sirotkin, A. V., Vyahhi, N., Tesler, G., Alekseyev, M. A., & Pevzner, P. A. (2012). SPAdes: A new genome assembly algorithm and its applications to single-cell sequencing. *Journal of Computational Biology*, *19*(5), 455–477. 10.1089/cmb.2012.0021 22506599 10.1089/cmb.2012.0021 PMC3342519

[CR3] Barclay, L., Davis, T., & Vinjé, J. (2021). Rare Norovirus GIV foodborne Outbreak, Wisconsin, USA. *Emerging Infectious Diseases*, *27*(4), 1151–1154. 10.3201/eid2704.204521 33754999 10.3201/eid2704.204521 PMC8007321

[CR4] Bolger, A. M., Lohse, M., & Usadel, B. (2014). Trimmomatic: A flexible trimmer for illumina sequence data. *Bioinformatics (Oxford England)*, *30*(15), 2114–2120. 10.1093/bioinformatics/btu170 24695404 10.1093/bioinformatics/btu170 PMC4103590

[CR5] Burlandy F. M., Malta F. C., Mello M. S., Fialho A. M., Baduy G. A., Guarnier R. V., Moreira K. C. S., & Fumian T. M (2025). Foodborne acute gastroenteritis outbreak associated with a rare norovirus recombinant GII.10[P16] genotype, Brazil, 2023. *120*(e0250085), 1–8. 10.1590/0074-02760250085 10.1590/0074-02760250085 PMC12622969 41259429

[CR6] Burnett, E., Parashar, U. D., & Tate, J. E. (2020). Global impact of rotavirus vaccination on diarrhea hospitalizations and deaths among Children < 5 years old: 2006–2019. *The Journal of Infectious Diseases*, *222*(10), 1731–1739. 10.1093/infdis/jiaa081 32095831 10.1093/infdis/jiaa081 PMC7483971

[CR7] Cantelli, C. P., Silva, M. R., Pimenta, L. M., Tavares, G. C. L., Baduy, G. A., Duch, A. A. S., Menezes, L. D. M., Fialho, A. M., Maranhão, A. G., Fumian, T. M., Miagostovich, M. P., & Leite, J. P. G. (2024). Evaluation of extraction methods to detect Noroviruses in Ready-to-Eat Raw milk Minas artisanal cheese. *Food and Environmental Virology*, *16*(2), 188–199. 10.1007/s12560-024-09588-1 38441780 10.1007/s12560-024-09588-1

[CR8] Cao, M., Yuan, F., Ma, X., Ma, J., Ma, X., Chen, H., Zhang, W., Zhao, J., & Kuai, W. (2023). Surveillance of human group A rotavirus in Ningxia, China (2015–2021): Emergence and prevalence of G9P[8]-E2 and G3P[8]-E2 genotypes. *Infection Genetics and Evolution*, *113*, 105469. 10.1016/j.meegid.2023.105469 10.1016/j.meegid.2023.105469 37331499

[CR9] Cardemil, C. V., Cortese, M. M., Medina-Marino, A., Jasuja, S., Desai, R., Leung, J., Rodriguez-Hart, C., Villarruel, G., Howland, J., Quaye, O., Tam, K. I., Bowen, M. D., Parashar, U. D., & Gerber, S. I. & the Rotavirus Investigation Team*. (2012). Two rotavirus outbreaks caused by genotype G2P[4] at large retirement communities: Cohort studies. Annals of Internal Medicine, 157(9), 621–631. 10.7326/0003-4819-157-9-201211060-00006 10.7326/0003-4819-157-9-201211060-00006 23128862

[CR10] Carvalho-Costa, F. A., de Assis, R. M. S., Fialho, A. M., Araújo, I. T., Silva, M. F., Gómez, M. M., Andrade, J. S., Rose, T. L., Fumian, T. M., Volotão, E. M., Miagostovich, M. P., & Leite, J. P. G. (2019). The evolving epidemiology of rotavirus A infection in Brazil a decade after the introduction of universal vaccination with Rotarix^®^. *BMC Pediatrics*, *19*(1), 42. 10.1186/s12887-019-1415-9 30704518 10.1186/s12887-019-1415-9 PMC6354375

[CR11] Cates, J. E., Amin, A. B., Tate, J. E., Lopman, B., & Parashar, U. (2021). Do rotavirus strains affect vaccine effectiveness? A systematic review and Meta-analysis. *The Pediatric Infectious Disease Journal*, *40*(12), 1135–1143. 10.1097/INF.0000000000003286 34870393 10.1097/INF.0000000000003286 PMC8966741

[CR12] Chen, S., Zhou, Y., Chen, Y., & Gu, J. (2018). Fastp: An ultra-fast all-in-one FASTQ preprocessor. *Bioinformatics (Oxford England)*, *34*(17), i884–i890. 10.1093/bioinformatics/bty560 30423086 10.1093/bioinformatics/bty560 PMC6129281

[CR13] Cohen, A. L., Platts-Mills, J. A., Nakamura, T., Operario, D. J., Antoni, S., Mwenda, J. M., Weldegebriel, G., Rey-Benito, G., de Oliveira, L. H., Ortiz, C., Daniels, D. S., Videbaek, D., Singh, S., Njambe, E., Sharifuzzaman, M., Grabovac, V., Nyambat, B., Logronio, J., Armah, G., & Serhan, F. (2022). Aetiology and incidence of diarrhoea requiring hospitalisation in children under 5 years of age in 28 low-income and middle-income countries: Findings from the global pediatric diarrhea surveillance network. *BMJ Global Health*, *7*(9), e009548. 10.1136/bmjgh-2022-009548 36660904 10.1136/bmjgh-2022-009548 PMC9445824

[CR14] Cowley, D., Donato, C. M., Roczo-Farkas, S., & Kirkwood, C. D. (2016). Emergence of a novel equine-like G3P[8] inter-genogroup reassortant rotavirus strain associated with gastroenteritis in Australian children. *The Journal of General Virology*, *97*(2), 403–410. 10.1099/jgv.0.000352 26588920 10.1099/jgv.0.000352

[CR15] Crawford, S. E., Ramani, S., Tate, J. E., Parashar, U. D., Svensson, L., Hagbom, M., Franco, M. A., Greenberg, H. B., O’Ryan, M., Kang, G., Desselberger, U., & Estes, M. K. (2017). Rotavirus infection. *Nature Reviews Disease Primers*, *3*, 17083. 10.1038/nrdp.2017.83 29119972 10.1038/nrdp.2017.83 PMC5858916

[CR16] Dóró, R., László, B., Martella, V., Leshem, E., Gentsch, J., Parashar, U., & Bányai, K. (2014). Review of global rotavirus strain prevalence data from six years post vaccine licensure surveillance: Is there evidence of strain selection from vaccine pressure? *Infection Genetics and Evolution: Journal of Molecular Epidemiology and Evolutionary Genetics in Infectious Diseases*, *28*, 446–461. 10.1016/j.meegid.2014.08.017 25224179 10.1016/j.meegid.2014.08.017 PMC7976110

[CR17] Esona, M. D., & Gautam, R. (2015). Rotavirus. *Clinics in Laboratory Medicine*, *35*(2), 363–391. 10.1016/j.cll.2015.02.012 26004648 10.1016/j.cll.2015.02.012

[CR18] Fumian, T. M., Ferreira, F. C., de Andrade, J., da Canal, S. R., Silva Gomes, N., Teixeira, G., L. B., & Miagostovich, M. P. (2021). Norovirus foodborne outbreak associated with the consumption of ice Pop, Southern Brazil, 2020. *Food and Environmental Virology*, *13*(4), 553–559. 10.1007/s12560-021-09495-9 34351587 10.1007/s12560-021-09495-9

[CR19] Gentsch, J. R., Glass, R. I., Woods, P., Gouvea, V., Gorziglia, M., Flores, J., Das, B. K., & Bhan, M. K. (1992). Identification of group A rotavirus gene 4 types by polymerase chain reaction. *Journal of Clinical Microbiology*, *30*(6), 1365–1373. 10.1128/jcm.30.6.1365-1373.1992 1320625 10.1128/jcm.30.6.1365-1373.1992 PMC265294

[CR20] Guerra, S. F. S., Soares, L. S., Lobo, P. S., Penha Júnior, E. T., Júnior, S., Bezerra, E. C., Vaz, D. A. M., Linhares, L. R., A. C., & Mascarenhas, J. D. P. (2016). Detection of a novel equine-like G3 rotavirus associated with acute gastroenteritis in Brazil. *The Journal of General Virology*, *97*(12), 3131–3138. 10.1099/jgv.0.000626 27902376 10.1099/jgv.0.000626

[CR21] Gutierrez, M. B., Arantes, I., Bello, G., Berto, L. H., Dutra, L. H., Kato, R. B., & Fumian, T. M. (2024). Emergence and dissemination of equine-like G3P[8] rotavirus A in Brazil between 2015 and 2021. *Microbiology Spectrum*, *12*(4), e0370923. 10.1128/spectrum.03709-23 38451227 10.1128/spectrum.03709-23 PMC10986506

[CR22] Gutierrez, M. B., Fialho, A. M., Maranhão, A. G., Malta, F. C., de Andrade, J. S. R., de Assis, R. M. S., Mouta, S., da Miagostovich, S. E., Leite, M. P., J. P. G., & Machado Fumian, T. (2020). Rotavirus A in brazil: Molecular epidemiology and surveillance during 2018–2019. *Pathogens (Basel Switzerland)*, *9*(7), 515. 10.3390/pathogens9070515 32605014 10.3390/pathogens9070515 PMC7400326

[CR23] Hill, V. R., Mull, B., Jothikumar, N., Ferdinand, K., & Vinjé, J. (2010). Detection of GI and GII Noroviruses in ground water using ultrafiltration and TaqMan Real-time RT-PCR. *Food and Environmental Virology*, *2*(4), 218–224. 10.1007/s12560-010-9049-y

[CR24] Iijima, Y., Iwamoto, T., Nukuzuma, S., Ohishi, H., Hayashi, K., & Kobayashi, N. (2006). An outbreak of rotavirus infection among adults in an institution for rehabilitation: Long-term residence in a closed community as a risk factor for rotavirus illness. *Scandinavian Journal of Infectious Diseases*, *38*(6–7), 490–496. 10.1080/00365540500532134 16798700 10.1080/00365540500532134

[CR25] ISO 15216-1:2017. (2017). Microbiology of food a chain—horizontal method for determination of Hepatitis A virus and norovirus in food using real-time RT-PCR—Part 1: Method for quantification. *International Organization for Standardization*, 2017, 1–48.

[CR27] Iturriza-Gómara, M., Isherwood, B., Desselberger, U., & Gray, J. (2001). Reassortment in vivo: Driving force for diversity of human rotavirus strains isolated in the united Kingdom between 1995 and 1999. *Journal of Virology*, *75*(8), 3696–3705. 10.1128/JVI.75.8.3696-3705.2001 11264359 10.1128/JVI.75.8.3696-3705.2001 PMC114861

[CR26] Iturriza Gómara, M., Wong, C., Blome, S., Desselberger, U., & Gray, J. (2002). Molecular characterization of VP6 genes of human rotavirus isolates: Correlation of genogroups with subgroups and evidence of independent segregation. *Journal of Virology*, *76*(13), 6596–6601. 10.1128/jvi.76.13.6596-6601.2002 12050372 10.1128/JVI.76.13.6596-6601.2002 PMC136279

[CR28] Kageyama, T., Kojima, S., Shinohara, M., Uchida, K., Fukushi, S., Hoshino, F. B., Takeda, N., & Katayama, K. (2003). Broadly reactive and highly sensitive assay for Norwalk-like viruses based on real-time quantitative reverse transcription-PCR. *Journal of Clinical Microbiology*, *41*(4), 1548–1557. 10.1128/JCM.41.4.1548-1557.2003 12682144 10.1128/JCM.41.4.1548-1557.2003 PMC153860

[CR29] Karataş, M., Bloemen, M., Cuypers, L., Wollants, E., Van Ranst, M., & Matthijnssens, J. (2025). 14 years of rotavirus A surveillance: Unusual dominance of equine-like G3P[8] genotype with DS-1-like genotype constellation after the pandemic, Belgium, 2009 to 2023. *Eurosurveillance*, *30*(12), 2400442. 10.2807/1560-7917.ES.2025.30.12.2400442 40156349 10.2807/1560-7917.ES.2025.30.12.2400442 PMC11951416

[CR30] Komoto, S., Tacharoenmuang, R., Guntapong, R., Ide, T., Tsuji, T., Yoshikawa, T., Tharmaphornpilas, P., Sangkitporn, S., & Taniguchi, K. (2016). Reassortment of human and animal rotavirus gene segments in emerging DS-1-Like G1P[8] rotavirus strains. *PloS One*, *11*(2), e0148416. 10.1371/journal.pone.0148416 26845439 10.1371/journal.pone.0148416 PMC4742054

[CR31] Lanzarini, N. M., Mannarino, C. F., Ribeiro, A. V. C., Prado, T., Vahia, L. S., Siqueira, M. M., Resende, P. C., Quintaes, B. R., & Miagostovich, M. P. (2023). SARS-CoV-2 surveillance-based on municipal solid waste leachate in Brazil. *Environmental Science and Pollution Research International*, *30*(25), 67368–67377. 10.1007/s11356-023-27019-9 37101215 10.1007/s11356-023-27019-9 PMC10132925

[CR32] Lewandowski, K., Xu, Y., Pullan, S. T., Lumley, S. F., Foster, D., Sanderson, N., Vaughan, A., Morgan, M., Bright, N., Kavanagh, J., Vipond, R., Carroll, M., Marriott, A. C., Gooch, K. E., Andersson, M., Jeffery, K., Peto, T. E. A., Crook, D. W., Walker, A. S., & Matthews, P. C. (2019). Metagenomic nanopore sequencing of influenza virus direct from clinical respiratory samples. *Journal of Clinical Microbiology*, *58*(1), e00963–e00919. 10.1128/JCM.00963-19 31666364 10.1128/JCM.00963-19 PMC6935926

[CR33] Lu, J., Rincon, N., Wood, D. E., Breitwieser, F. P., Pockrandt, C., Langmead, B., Salzberg, S. L., & Steinegger, M. (2022). Metagenome analysis using the kraken software suite. *Nature Protocols*, *17*(12), 2815–2839. 10.1038/s41596-022-00738-y 36171387 10.1038/s41596-022-00738-y PMC9725748

[CR34] Luchs, A., Da Costa, A. C., Cilli, A., Komninakis, S. C. V., Carmona, R. D. C. C., Boen, L., Morillo, S. G., Sabino, E. C., & Timenetsky, M. D. C. S. T (2019). Spread of the emerging equine-like G3P[8] DS-1-like genetic backbone rotavirus strain in Brazil and identification of potential genetic variants. *Journal of General Virology*, *100*(1), 7–25. 10.1099/jgv.0.001171 30457517 10.1099/jgv.0.001171

[CR35] Ma, J., Kumbhakar, R. G., Casto, A., Chow, E. J., Englund, J. A., Gautam, R., Jaimes, J., Tate, J. E., Smart, S., Mani, N. S., Cohen, S. A., Hussein, A., Rietberg, K., Bryson-Cahn, C., & Fang, F. C. (2025). Outbreak of rotavirus diarrheal infection among adults in King County, Washington, January–June 2023. *The Journal of Infectious Diseases*, *231*(6), 1553–1558. 10.1093/infdis/jiaf013 39774704 10.1093/infdis/jiaf013

[CR36] Malasao, R., Saito, M., Suzuki, A., Imagawa, T., Nukiwa-Soma, N., Tohma, K., Liu, X., Okamoto, M., Chaimongkol, N., Dapat, C., Kawamura, K., Kayama, Y., Masago, Y., Omura, T., & Oshitani, H. (2015). Human G3P[4] rotavirus obtained in Japan, 2013, possibly emerged through a human–equine rotavirus reassortment event. *Virus Genes*, *50*(1), 129–133. 10.1007/s11262-014-1135-z 25352228 10.1007/s11262-014-1135-z PMC4349953

[CR37] Martin, M. (2011). Cutadapt removes adapter sequences from high-throughput sequencing reads. *EMBnet Journal*, *17*(1), 10. 10.14806/ej.17.1.200

[CR38] Matthijnssens, J., Ciarlet, M., Heiman, E., Arijs, I., Delbeke, T., McDonald, S. M., Palombo, E. A., Iturriza-Gómara, M., Maes, P., Patton, J. T., Rahman, M., & Van Ranst, M. (2008a). Full genome-based classification of rotaviruses reveals a common origin between human Wa-Like and Porcine rotavirus strains and human DS-1-like and bovine rotavirus strains. *Journal of Virology*, *82*(7), 3204–3219. 10.1128/JVI.02257-07 18216098 10.1128/JVI.02257-07 PMC2268446

[CR40] Matthijnssens, J., Ciarlet, M., Rahman, M., Attoui, H., Bányai, K., Estes, M. K., Gentsch, J. R., Iturriza-Gómara, M., Kirkwood, C. D., Martella, V., Mertens, P. P. C., Nakagomi, O., Patton, J. T., Ruggeri, F. M., Saif, L. J., Santos, N., Steyer, A., Taniguchi, K., Desselberger, U., & Van Ranst, M. (2008b). Recommendations for the classification of group A rotaviruses using all 11 genomic RNA segments. *Archives of Virology*, *153*(8), 1621–1629. 10.1007/s00705-008-0155-1 18604469 10.1007/s00705-008-0155-1 PMC2556306

[CR39] Matthijnssens, J., Ciarlet, M., McDonald, S. M., Attoui, H., Bányai, K., Brister, J. R., Buesa, J., Esona, M. D., Estes, M. K., Gentsch, J. R., Iturriza-Gómara, M., Johne, R., Kirkwood, C. D., Martella, V., Mertens, P. P. C., Nakagomi, O., Parreño, V., Rahman, M., Ruggeri, F. M., & Van Ranst, M. (2011). Uniformity of rotavirus strain nomenclature proposed by the rotavirus classification working group (RCWG). *Archives of Virology*, *156*(8), 1397–1413. 10.1007/s00705-011-1006-z 21597953 10.1007/s00705-011-1006-z PMC3398998

[CR41] Medici, M. C., Tummolo, F., Martella, V., Arcangeletti, M. C., De Conto, F., Chezzi, C., Magrì, A., Fehér, E., Marton, S., Calderaro, A., & Bányai, K. (2016). Whole genome sequencing reveals genetic heterogeneity of G3P[8] rotaviruses circulating in Italy. *Infection Genetics and Evolution*, *40*, 253–261. 10.1016/j.meegid.2016.03.013 10.1016/j.meegid.2016.03.013 26980605

[CR42] Morozova, O. V., Sashina, T. A., Epifanova, N. V., Velikzhanina, E. I., & Novikova, N. A. (2023). Phylodynamic characteristics of reassortant DS-1-like G3P[8]-strains of rotavirus type A isolated in Nizhny Novgorod (Russia). *Brazilian Journal of Microbiology*, *54*(4), 2867–2877. 10.1007/s42770-023-01155-3 37897627 10.1007/s42770-023-01155-3 PMC10689624

[CR43] Niendorf, S., Ebner, W., Marques, A. M., Bierbaum, S., Babikir, R., Huzly, D., Maaßen, S., Grundmann, H., & Panning, M. (2020). Rotavirus outbreak among adults in a university hospital in Germany. *Journal of Clinical Virology*, *129*, 104532. 10.1016/j.jcv.2020.104532 32650277 10.1016/j.jcv.2020.104532

[CR44] Omatola, C. A., & Olaniran, A. O. (2022). Rotaviruses: From pathogenesis to disease control—A. *Critical Review Viruses*, *14*(5), 875. 10.3390/v14050875 35632617 10.3390/v14050875 PMC9143449

[CR45] Pietsch, C., & Liebert, U. G. (2018). Molecular characterization of different equine-like G3 rotavirus strains from Germany. *Infection Genetics and Evolution*, *57*, 46–50. 10.1016/j.meegid.2017.11.007 10.1016/j.meegid.2017.11.007 29128517

[CR46] Pintó, R. M., Costafreda, M. I., & Bosch, A. (2009). Risk assessment in shellfish-borne outbreaks of hepatitis A. *Applied and Environmental Microbiology*, *75*(23), 7350–7355. 10.1128/AEM.01177-09 19820160 10.1128/AEM.01177-09 PMC2786421

[CR47] Rajal, V. B., McSwain, B. S., Thompson, D. E., Leutenegger, C. M., Kildare, B. J., & Wuertz, S. (2007). Validation of Hollow fiber ultrafiltration and real-time PCR using bacteriophage PP7 as surrogate for the quantification of viruses from water samples. *Water Research*, *41*(7), 1411–1422. 10.1016/j.watres.2006.12.034 17313967 10.1016/j.watres.2006.12.034

[CR48] Raymond, P., Paul, S., Perron, A., Bellehumeur, C., Larocque, É., & Charest, H. (2022). Detection and sequencing of multiple human Norovirus genotypes from imported frozen raspberries linked to outbreaks in the Province of Quebec, Canada, in 2017. *Food and Environmental Virology*, *14*(1), 40–58. 10.1007/s12560-021-09507-8 35066807 10.1007/s12560-021-09507-8 PMC8881426

[CR49] Rumble, C., Addiman, S., Balasegaram, S., Chima, K., Ready, D., Heard, J., & Alexander, E. (2017). Role of food handlers in Norovirus outbreaks in London and South East England, 2013 to 2015. *Journal of Food Protection*, *80*(2), 257–264. 10.4315/0362-028X.JFP-16-083 28221985 10.4315/0362-028X.JFP-16-083

[CR50] Saupe, A. A., Rounds, J., Sorenson, A., Hedeen, N., Bagstad, E., Reinberg, R., Wagley, A. G., Cebelinski, E., & Smith, K. (2021). Outbreak of Norovirus gastroenteritis associated with ice cream contaminated by frozen raspberries from China—Minnesota, united States, 2016. *Clinical Infectious Diseases*, *73*(11), e3701–e3707. 10.1093/cid/ciaa821 32564069 10.1093/cid/ciaa821

[CR51] Troeger, C., Khalil, I. A., Rao, P. C., Cao, S., Blacker, B. F., Ahmed, T., Armah, G., Bines, J. E., Brewer, T. G., Colombara, D. V., Kang, G., Kirkpatrick, B. D., Kirkwood, C. D., Mwenda, J. M., Parashar, U. D., Petri, W. A., Riddle, M. S., Steele, A. D., Thompson, R. L., & Reiner, R. C. (2018). Rotavirus vaccination and the global burden of rotavirus diarrhea among children younger than 5 years. *JAMA Pediatrics*, *172*(10), 958–965. 10.1001/jamapediatrics.2018.1960 30105384 10.1001/jamapediatrics.2018.1960 PMC6233802

[CR52] Umair, M., Abbasi, B. H., Sharif, S., Alam, M. M., Rana, M. S., Mujtaba, G., Arshad, Y., Fatmi, M. Q., & Zaidi, S. Z. (2018). High prevalence of G3 rotavirus in hospitalized children in Rawalpindi, Pakistan during 2014. *PLOS ONE*, *13*(4), e0195947. 10.1371/journal.pone.0195947 29708975 10.1371/journal.pone.0195947 PMC5927433

[CR53] Vizzi, E., Rosales, R. E., Piñeros, O., Fernández, R., Inaty, D., López, K., Peña, L., De Freitas-Linares, A., Navarro, D., Neri, S., Durán, O., & Liprandi, F. (2025). Emergence of Equine-like G3P[8] rotavirus strains infecting children in Venezuela. *Viruses*, *17*(3), 410. 10.3390/v17030410 40143336 10.3390/v17030410 PMC11946648

[CR54] Wang, Y., Li, J., Liu, P., & Zhu, F. (2021). The performance of licensed rotavirus vaccines and the development of a new generation of rotavirus vaccines: A review. *Human Vaccines & Immunotherapeutics*, *17*(3), 880–896. 10.1080/21645515.2020.1801071 32966134 10.1080/21645515.2020.1801071 PMC7993146

[CR55] Wikswo, M. E., Roberts, V., Marsh, Z., Manikonda, K., Gleason, B., Kambhampati, A., Mattison, C., Calderwood, L., Balachandran, N., Cardemil, C., & Hall, A. J. (2022). Enteric illness outbreaks reported through the National outbreak reporting System—United States, 2009–2019. *Clinical Infectious Diseases*, *74*(11), 1906–1913. 10.1093/cid/ciab771 34498027 10.1093/cid/ciab771 PMC11194694

[CR56] Wood, D. E., Lu, J., & Langmead, B. (2019). Improved metagenomic analysis with kraken 2. *Genome Biology*, *20*(1), 257. 10.1186/s13059-019-1891-0 31779668 10.1186/s13059-019-1891-0 PMC6883579

[CR57] World Health Organization. (‎2009)‎. Rotavirus vaccines: An update. Weekly Epidemiological Record = Relevé épidémiologique hebdomadaire, 84 (‎51–52)‎, 533–540. https://iris.who.int/items/d726f9bb-af56-4f2a-a2b2-de4a6629ffc4. 20034143

[CR58] Zeng, S. Q., Halkosalo, A., Salminen, M., Szakal, E. D., Puustinen, L., & Vesikari, T. (2008). One-step quantitative RT-PCR for the detection of rotavirus in acute gastroenteritis. *Journal of Virological Methods*, *153*(2), 238–240. 10.1016/j.jviromet.2008.08.004 18765254 10.1016/j.jviromet.2008.08.004

